# The Construction of Psychological Intervention Mechanism of Deep Learning in the Prevention of Legal Anomie

**DOI:** 10.3389/fpsyg.2022.937268

**Published:** 2022-09-05

**Authors:** Caixia Zou

**Affiliations:** Shanghai University of Political Science and Law, Shanghai, China

**Keywords:** deep learning, legal anomie, psychological intervention, particularly, algorithm

## Abstract

The convenience of big data processing technology has played a great advantage in many scenarios, and its deep learning can effectively mine different types of data in data sets. Applying this method to mining psychological prediction data set of legal anomie behavior can effectively prevent the occurrence of illegal behavior. The effective analysis of its psychological characteristics and the changes of psychological emotions will have hidden dangers, so it is necessary to extract this kind of data in such cases. Therefore, the forest algorithm under deep learning uses weight factors, simple sampling, and other ideas to build the intervention mechanism, which will improve psychological quality to prevent anomie. The experimental results of this paper are as follows: (1) In the case of testing psychological fluctuation by three algorithms, the forest algorithm calculates the psychological change area steadily, with the increase of the number of samples. (2) In the data sample extraction of different dimensions, the test value increases with the increase of dimensions, and it is stable at 12 dimensions. (3) Preventive intervention is extremely important to construct the mechanism before the anomie behavior occurs, and the longest running time of the roll neural network is as much as 950 s. (4) The data mining technology for constructing the intervention mechanism accounts for 15.4% of counseling, which also shows that the auxiliary measures for preventing behavior are particularly important.

## Introduction

With the rapid development of data acquisition technology, most researchers will mine important information as the foundation, which is the importance of in-depth research. In the modern era of severe punishment by law, some bad habits must be standardized. Before people violate them, their psychology will fluctuate obviously, and their characteristics will also be manifested. This paper is to explore a new research field to extract key information to retrieve, if there are anomalies, it needs to be optimized. The basic mechanism is constructed by fast mining, high efficiency, high quality, cluster analysis, and other analysis methods. When it is applied to the decision analysis of psychological changes, preventive measures can be carried out, which greatly reduces the probability of crime. This paper studies the phenomenon of college students’ moral anomie, and puts forward some countermeasures to correct the moral anomie of college students (Zhou, 2019). The theory of “quality difference” is used to reclassify the acts of violating public security administration and criminal acts, and to allocate punishment measures in line with the characteristics of their respective sanction systems (Jin, 2016). It determines that judicature needs to use qualitative differences and quantitative differences according to the nature of infringement of legal interests so as to realize effective distinction and convergence of laws (Sun, 2017). This article intends to observe the effect of psychological intervention on COPD patients with dyspnea, indicating that this may be related to the anxiety state of patients (Sun et al., 2014). It studies the effect of focused psychological intervention (FPI) on self-management ability, and analyzes it by an comparative experiment between the control group and the routine group (He et al., 2020). It discusses the intervention effect of group psychological counseling combined with peer education on depression, and there is no statistical difference in anxiety and depression scores (Sun et al., 2016). This paper evaluates the ability of psychological crisis intervention in public emergencies, and provides a theoretical basis for future psychological crisis intervention in public emergencies (Chen et al., 2013). It analyzes the psychological problems and characteristics of different groups, such as professional earthquake rescue team members, emergency rescue personnel, and earthquake rescue volunteers at the earthquake site (Zhang and Sun, 2013). It expounds the main psychological reactions of post-traumatic adolescents and the theoretical basis of their research, and summarizes the status and development trend of post-traumatic stress disorder, depression, and post-traumatic growth of adolescents (Wu et al., 2015). Using canonical correlation analysis, it is found that there is a positive correlation between academic ecological environment and academic anomie behavior tendency (Li and Shang, 2017). It explores the reasons for teenagers’ network moral anomie and puts forward the characteristics of virtuality and concealment of cyberspace (Li, 2019). Using SCL-90 to investigate the mental health status of community medical staff at the baseline and evaluate the effect of psychological intervention measures (Huang and Xi, 2018). It discusses the effect of group psychological counseling on its intervention and investigates the current situation of psychiatrists’ work stress (Zhao and Gong, 2016). It analyzes the mental health status of patients with occupational disease and discusses the effect of psychological intervention measures (Huang et al., 2013). To explore the effect of comprehensive psychological intervention on the psychological behavior of patients with depression, a convenient sampling method was used to investigate the psychological patients (Jiang et al., 2013). The second part of the article introduces the theory and the method of deep learning, the third part studies the weight of random forest algorithm in depth, and discusses the psychological adaptive method. The fourth part compares and analyzes the construction of the psychological intervention mechanism of deep learning in the prevention of legal anomie.

## Deep Learning Algorithm Theory and the Accelerated Learning Method

### Basic Theory of Deep Learning Algorithm

**FIGURE 1:**
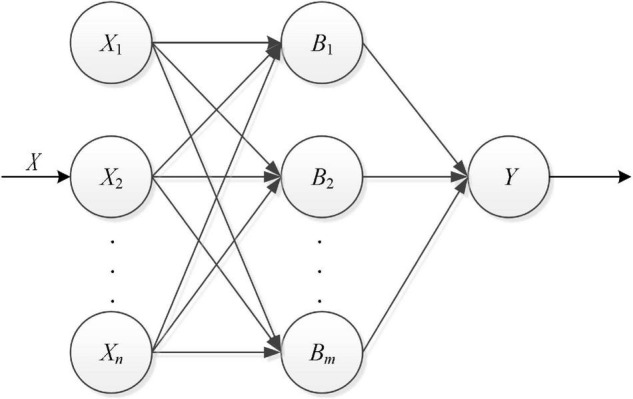
Shallow fully connected neural network.

With the continuous maturity of artificial intelligence technology, deep learning has been widely used in various fields, and its network structure has evolved from the initial shallow layer to the deep layer. Typically, the input and output values are classified and calculated by shallow fully connected neural networks, and the hierarchical complexity is judged by the correlation of neural nodes under the same hierarchy. The network diagram looks like this; it is shown in Figure 1.

The physical meaning of each layer under the network level is expressed according to its unique characteristics, and there is only one hidden layer in the shallow layer and no more than two hidden layers. However, in the deep network structure, there will be more layers and stronger expression ability, and the product of width and depth will obtain more network resources. No matter what kind of model, it should train and calculate the sample data set to improve the recognition and security.

(1) There are too many model parameters in deep learning, and the complex algorithm requires too-high computing power of hardware.
(2) It is difficult to obtain data, which require a lot of experiments, and the time front is too long.
(3) The selected hierarchical convolution is difficult to explain semantics, which leads to slow improvement progress.
(4) Due to the problem of fitting relationship, the model has complex non-linear relationship and lacks high performance.

### Heterogeneous Accelerated Design of Convolution Neural Network

Inspired by the biological neuron model and the recognition process of things, the artificial neural network is designed. The network has the characteristics of self-learning and non-linearity in the data set, which can identify the forward and reverse transmission of data. Its structure consists of three hierarchical structures, which can reduce the complexity of the model and avoid the failure of fitting results. Its heterogeneous algorithm is as follows:

Maximum pooling calculation (Wen, 2012). The formula is as follows:


(1) $$x_{m\,j}^{l}=\frac{1}{n\times n}\,\sum x_{m\,j}^{l-1},x_{m\,j}^{l-1}\in V_{m\,j}$$

(2) $$x_{m\,j}^{l-1}=\max\left(x_{m\,l}^{l-1},\ldots ,x_{m\,n^{2}}^{l-1}\right),x_{m\,i}^{l-1}\in V_{m\,j}$$

In the formula, in *n*-pooled window widths, the output result of *x* is obtained, and *V* is the characteristic image of each plate.

The calculation of activation function is to stimulate the biological nerve, which includes the regression model of linear characteristics. This is the process of recombining linear functions to reduce the bias generated by each layer to smaller and smaller, and finally the gradient disappears.

Activate the function (Pan, 2017). Evaluate expression:


(3) $$f\,\left(x\right)=\frac{1}{1+e^{-x}}$$

In order to accelerate the convergence speed of the model training process, the problem of large amount of calculation and gradient diffuse are degraded. The calculation process is as follows:


(4) $$f\,\left(x\right)=\frac{e^{x}-e^{-x}}{e^{x}+e^{-x}}$$

When the input value is greater than 0, the output value is equal to the input value; when the input value is less than 0, the output value is all 0. This is related to biological excitement, and the next result will be that the activation function will not be updated. Its calculation is expressed as:


(5) f⁢(x)=max⁡(0,x)

The full connection layer is responsible for the extraction of integrated eigenvalues, and the classification of input data is convenient for regression analysis, so the number of parameters is dense, occupying the network space, and the calculation amount of the convolution layer is reduced. The calculation amount of the full connection layer is also reduced accordingly (Sun et al., 2014). The expression is:


(6) $$O\,f\,\left(o\right)=\sum_{f\,i=0}^{F\,n}I\,\left(f\,i\right)\times W\,\left(f\,o,f\,i\right)+B\,\left(f\,o\right)$$

where *I* represents the input value of the *fi* channel. *O* represents the output value of *fo*. The weights of each level are obtained through the calculation results of bias *B*, and the complex network model is obtained by connecting each feature point with weights. Finally, for the function range of control nodes, each parameter model is normalized. The processing algorithm is:

Normalization (Liang and Lin, 2016).


(7) $$G\,x\,\left(u\,i\right)=\frac{1}{\sum \begin{array}{c} k\\ j-1 \end{array}\,e\,x_{j}^{T}\,u_{i}}\,\left[\begin{array}{c} e\,x_{1}^{T}\,u_{i}\\ e\,x_{2}^{T}\,u\\ \vdots \\ e\,x_{k}^{T}\,u \end{array}\right]$$

Suppose there are *n* training sets, which are *u*_1_, *u*_2_, *u*_3_, …, *u*_*n*_, and their classification expression is *V*. For solving a certain class probability P of input, all output values are summed to 1 after normalization.

### Outlier Data Mining

Outlier data have different distribution ranges of features in the data set, which leads to a deviation phenomenon in the performance process. In view of this kind of situation, statistical classification calculation can be carried out according to the proportion weight of elements so as to make accurate judgment in the detection process. The weight calculation is as follows:


(8) $$W_{\left(i\right)}=\frac{P_{i}}{\sum \begin{array}{c} n\\ i=1 \end{array}\,P_{i}}$$

(9) $$W_{\left(i\right)}=\frac{1-E_{i}}{k-\sum E_{\_\,i}}$$

Pi represents the number of attribute samples, and *E*_*i*_ is the information entropy of each element attribute. The tangent correlation logic function of hyperbola is used as weighting function, and the discrete points of judging elements are used as data representation. The main method of parameter estimation in data mining is before and after distribution. The maximum likelihood estimation is used to estimate the parameters of the distribution (Pan, 2015). The formula is:


(10) $$I\,n\,L\,\left(\mu ,\sigma^{2}\right)=\sum_{i=1}^{n}I\,n\,f\,\left(x_{i}|\left(\mu ,\sigma^{2}\right)\right)$$

(11) $$\overset{\wedge }{\mu }=\frac{1}{n}\,\sum_{i=1}^{n}x_{i}$$

(12) $$\overset{\wedge^{2}}{\sigma }=\frac{1}{n}\,\sum_{i=1}^{n}{\left(x_{i}-\overset{-}{x}\right)}^{2}$$

Next, the node splitting index is calculated, and the attribute dimension is fitted. It adopts the principle of random selection for each child node by the decision tree method, and merges each decision with a split index in an attribute dimension. One of its data objects has different calculation paths in different decision trees, but it will plan the next level distribution according to the characteristic entropy.

Gini index (Shi and Lu, 2018). The calculation formula is:


(13) $$G=\sum \begin{array}{c} t\\ i=1 \end{array}\,P_{t}\,\left(1-P_{t}\right)$$

(14) $$G\,\left(D,A\right)=\frac{\left|D_{1}\right|}{\left|D\right|}\,G\,\left(D_{1}\right)+\frac{\left|D_{2}\right|}{\left|D\right|}\,G\,\left(D_{2}\right)$$

A is any attribute in the data set *D*, and the probability *Pt* of the T-class data set is calculated. According to the probability vectors, the lower vectors are combined, and the cross-validation method is used for training. Cross-validation value (Tian and Zhou, 2017). The formula is:


(15) $$\beta =\frac{\sum \begin{array}{c} n\\ i=1 \end{array}\,Q_{i}}{n}$$

where *n* is the number of subsets, and *Q*_*i*_ is the result of classification *i* times. The maximum number of connection layers is obtained through the iteration of each level of connection, and the maximum average value is used as the output value.

#### Data Mining Algorithm of Deep Forest Algorithm

The feature selection module under the cascade model is constructed, which is convenient for simplifying the problem description method and constructing the forest prediction probability matrix. Firstly, the cascade forest module is analyzed, and the features are randomly selected in the algorithm, and the accuracy rate is calculated by predicting the probability matrix. The higher the accuracy, the higher the correlation weight factor and the greater the weight, otherwise the smaller the weight. This step can effectively reduce the impact of feature random selection node performance.

Forest prediction probability matrix (Wang, 2017). The calculation formula is as follows:


(16) $$B=\left(\begin{array}{ccc} B_{11} & \ldots & B_{1\,n}\\ \vdots & \ddots & \vdots \\ B_{m\,1} & \cdots & B_{m\,n} \end{array}\right)$$

Where the data sets and categories are:


(17) $$D=\left\{L_{1},L_{2},\ldots ,L_{n}\right\}$$

(18) $$C=\left\{C_{1},C_{2},\ldots \,C_{n}\right\}$$

*B*_*ij*_ represents the classification probability of these data. According to the definition of the maximum value in the forest prediction matrix, the matrix is marked as 1, and the rest is 0, for example:


(19) $$B=\left(\begin{array}{ccc} 0 & \ldots & 1\\ \vdots & \ddots & \vdots \\ 1 & \cdots & 0 \end{array}\right)$$

Accuracy rate (Zhao, 2019). The formula is as follows:


(20) $$P=\frac{\sum_{i=0}^{m}\sum_{j=0}^{n}A\,\left[i\right]\,\left[j\right]\cap B\,\left[i\right]\,\left[j\right]}{m}$$

where m is the total number of data, *n* is the number of categories, the actual category matrix is solved for *A*, the prediction probability matrix is solved for *B*, and the accuracy rate of each predicted value is obtained through operation.

#### Definition of Weight Factor

In order to deepen the mining speed of weighted deep forest algorithm, it is necessary to define new factors. This operation is to filter out the eigenvalues of irrelevant attributes, which can effectively reduce the influence of irrelevant factors on the algorithm. Repeat the above operations until the maximum number of cascade layers is reached. Suppose the weight factor of the forest is *u* and defined as:


(21) $$\mu =\frac{\log_{2}P_{i}}{\sum \begin{array}{c} r\\ i=1 \end{array}\,\log_{2}P_{i}}$$

*P*_*i*_ represents the accuracy of *i* forests, and its matrix expression is:


(22) $$B_{\left(i\right)}=B\times \mu$$

In order to deepen the effective use of forest matrix in data mining, the isolation factor is defined again, and the specific expression forms are as follows:

With binary Group *D* as the background and *C* as the classification set, the isolation factor a can be defined as:


(23) $$M_{\left(L_{t}\right)}=\min\left\{d\,\left(L_{t},L_{1}\right),d\,\left(L_{t},L_{2}\right),\ldots ,d\,\left(L_{t},L_{n}\right)\right\}$$

(24) $$\rho_{\left(C_{i}\right)}=\frac{\sum \begin{array}{c} n\\ t=1 \end{array}\,M_{\left(L_{t}\right)}}{n}$$

(25) $$\alpha =\frac{\rho_{\left(C_{i}\right)}}{\sum \begin{array}{c} r\\ i=1 \end{array}\,\rho_{\left(C_{i}\right)}}$$

Where *d* represents the distance between data points and ρ represents the class density in the data, then the threshold function of the isolation factor is expressed as:

Threshold function (Yang, 2017).


(26) $$F\,\left(r\right)={\frac{1}{r}}^{*}\,\log_{10}r$$

Where *r* is the number of categories of the data set *r* > 0, and the threshold function *F* (*r*) decreases monotonously. In the construction process, due to the different distributions, it will be divided into categories with different densities. Through the class density of each type of sample data, more and more categories will be classified; otherwise, the density will decrease. Next, the isolated factor α is solved locally, and the greater the probability, the higher the threshold value of the obtained function will be.

## Insight Into the Environment of Weighted Forest Algorithm

### Analysis of Environmental Data Set of Anomie Behavior Formation

With the rapid development of modern society and economy, it is influenced by social transformation, the combination of Chinese and Western cultures, and the environmental changes, such as network technology. Nowadays, most people’s values are gradually changing and sublimating. With the increase of the consumption level, driven by the temptation of money, a few people will take the road of breaking the law and committing crimes. This is the basic condition that the change of economic environment leads people to violate the bottom line of the law, which will lead to the breaking of the basic norms of society and the infinitely enlarged road of individual crime.

Taking the anomie behavior caused by network consumption as an example, its network consumption has become a common consumption mode, and some criminals use false information to commit fraud against people, resulting in improper economic transactions. The power and convenience of the Internet make people’s consumption patterns, and heights have undergone qualitative changes. Some old people who are not aware of self-prevention are easily fooled, so legal anomie will be fully manifested in the network environment. Inciting emotions and distorting values in the network environment often lead to irrational behaviors, which fall into the illegal road. Preventive measures should be taken in time to minimize injuries and economic losses. Driven by certain interests, some activities violate the norms and requirements of the law, deviate from the normal running track of society, and destroy the public order. This not only damages the image and reputation of individuals but also has a great impact on normal life. Therefore, in the whole public security management, more control and comprehensive management should be taken.

### An Analysis of the Motivation of the Formation of the Self-Psychological Data Set

#### Internal Cause Analysis

The internal cause of anomie behavior is that human beings cannot control their desires when facing temptation and do some things that violate moral laws and regulations. Unable to restrain inner curiosity, face desire, and bear psychological pressure are all the reasons for making mistakes. When pursuing money and status, they often cannot control their mood and use some illegal means to obtain their own interests and bring harm to others. Therefore, in the state of mind, we should always keep a kind heart to repay the society, pursue the state of mind of self-sublimation, not rely on money to do face-saving projects, and improve our ability to resist temptation in time.

#### External Cause Analysis

Facing the modern society with free life and open mind, people’s moral quality has been gradually improved, and the good social atmosphere of civilization, law-abiding, and order maintenance has been fully carried out. The control measures introduced by relevant departments will greatly restrict people from doing some morally corrupt things to a good society. The government should issue relevant policies in time, strengthen the management of moral construction, and let people know from their bones that they have the habit of abiding by laws and regulations.

As a potential educational organization of moral anomie, schools should give legal education and guidance to students in the enlightenment stage. Some criminals do not act in the stage of education habits, which eventually leads to the lack of the minimum knowledge of obeying laws and regulations in psychology. Before being exposed to the social atmosphere and rules, high efficiency is the basic environmental condition for students to learn how to be a man and have good habits. On the contrary, it lacks the reserve of legal knowledge when it pays attention to learning, and it also fails to cultivate a good and useful talent. Through habits, life and the information that online students can come into contact with, they can learn the ability to distinguish right from wrong, which can strengthen people’s autonomy.

Finally, the cause of the whole data set is tested, and the test value is close to 1 every time. Its data cause set algorithm is:


(27) $$I\,\left(P_{1},P_{0}\right)=\left\{ \begin{array}{c} 1,P_{1}>P_{0}\\ 0.5,P_{1}=P_{0}\\ 0,P_{1}<P_{0} \end{array}\right.$$

*P*_1_ represents the normal prediction probability of samples, and *P*_0_ is the abnormal data set. The number of internal and external data sets, that is, the whole curve area can be defined to the vicinity of the test value 1, which can effectively improve the detection effect. Based on the experimental concept of integral integration, the experiment extracts and calculates multi-type data sets in the set. According to the evaluation standard of the psychological intervention mechanism, the overall algorithm performance is relatively stable and centralized. With the increase of the whole data set, the changes of the algorithm in solving psychological emotions will also increase.

### Countermeasure Development Under Deep Forest

**FIGURE 2:**
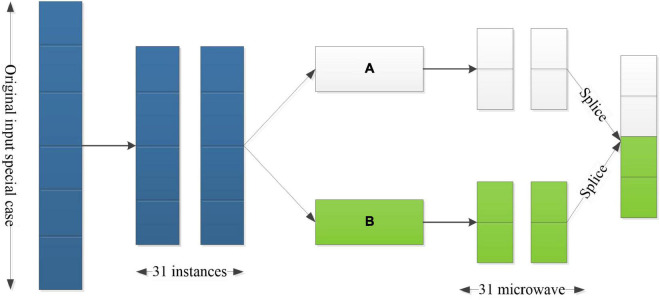
A schematic diagram of multi-scan structure.

For each individual population, anomie behavior may occur. In order to reduce the proportion of errors in this situation, the population can be selected two times, that is, the data entering random forest and the training of complete forest algorithm are combined. Vector mosaic for the probability that this kind of situation may occur is to transform the whole data set into a probability arrangement matrix. According to the window experimental distribution diagram, it is arranged as follows:

It can be seen from the Figure 2 that, after inputting the original features, 31 instances are screened two times according to the post-processing, and the other two instance results are obtained. After each special case has obvious differences, the diversity and consistency of the process can be guaranteed. Therefore, the converted subsampling is, actually, a half-and-half processing of data so as to reduce the dimensions between variables and achieve accurate selection of node eigenvalues.

(1) The input feature is the first random sampling based on 100-dimensional basic conditions.
(2) When the size of the tap is 40, 31 feature options are effectively obtained.
(3) Carry out secondary partition, taking A and B as subsets.
(4) Finally, the instances are pieced together, and the transformed instances are pieced together as feature vectors.

### The Weighted Fast Construction Method of Forest Algorithm

After rapid feature extraction based on multiple sampling, the discrete points are detected, and the converted data are analyzed according to the window size. The probability vector will be defined in the cascade module of the input value, and the accuracy will be calculated so that the probability vector can enter the next level of iteration. The specific construction method is shown as Algorithm 1:

**ALGORITHM 1:** The specific construction method.

Input: Training Set *D*, total number of samples *X* and number of categories *R*
Output: Discrete point data
1. For *r* = 1 to *R* do 2. Calculate the class density ρ(*C*_*i*_) 3. Calculate the isolation factor alpha 4. End for 5. Calculate the threshold function *F* (*R*)

After rapid construction of weighted depth forest based on multiple sampling, the scanning time of a large number of training data can be reduced, thus reducing the complexity of time. In this way, the depth algorithm constructed by weighting can enlarge the window of the overall trend in performance, which will improve the quality of later calculation and decision-making.

## Construction of the Psychological Budget Law Mechanism Before Facing Anomie Behavior

### Psychological Change Curves of Various Algorithms Before Behavior

**FIGURE 3:**
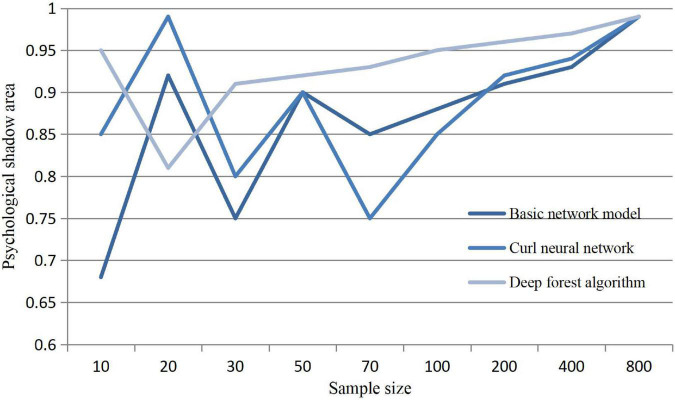
Influence of sample size on psychological changes.

According to the research, when people commit some illegal acts, they often have psychological and emotional fluctuations, so they can do a good job of behavioral care in time to prevent the occurrence of illegal acts. In order to intuitively see the fluctuation of psychological emotions, we analyze and deal with the construction of various algorithms in the intervention mechanism. The result curve is as follows:

As can be seen from Figure 3, with the increase of the number of data sets in the prediction calculation under each algorithm, people’s anomie psychology will increase. In the case of too many people, people’s criminal psychological activities will be greatly improved, and the occurrence of herd psychology will lead to wrong judgment. Deep forest calculation in the performance test can effectively and smoothly calculate sample measurements.

### Analysis of the Influence of Different Dimensions on Psychological Intervention

**FIGURE 4:**
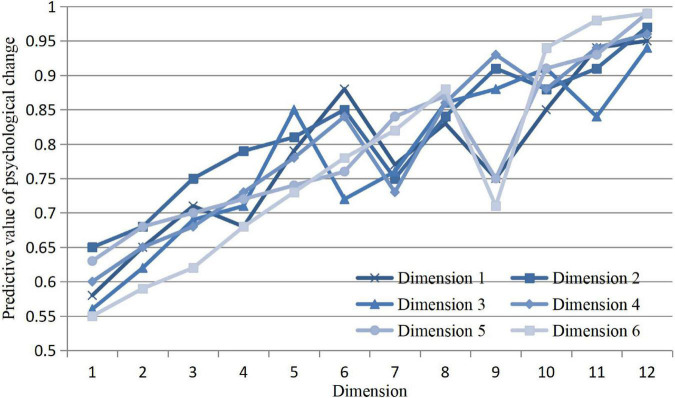
Prediction results of psychological intervention under different dimensions.

Algorithms in different dimensions may have obvious differences in prevention results before behavior occurs. In order to understand the prediction accuracy of various algorithms in different dimensions more clearly, the psychological changes are judged by six dimension values. The dimension will be tested with 1–12 weights, and the results are as follows in Figure 4.

According to the prediction results, the prediction results in different dimensions will interfere with psychological prevention. Under the calculation results of Dimensions 1, 2, and 3, it will gradually increase with the increase of dimensions; the prediction results in Dimensions 5, 6, and 7 will lead to large fluctuations. Especially when the dimension is 12, its convergence value will be infinitely close to the maximum value of 1, which can ensure the high efficiency of the algorithm when the dimension is 12.

### Analysis of Deep Learning Predicting Running Time of Psychological Behavior

**FIGURE 5:**
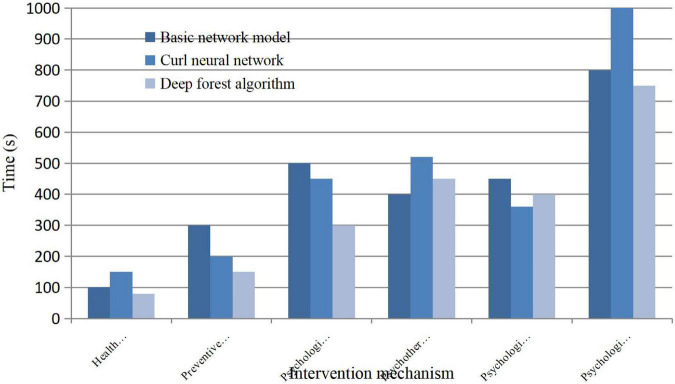
Comparison results of predicted runtime.

Modular feature transformation needs to be completed in a certain time, and the algorithm error is reduced by the weight factor in data mining technology. That is to say, the detection and analysis of the psychological status data set can be carried out, and the quality of mining will be detected by time comparison, which is as follows:

Figure 5 shows the data results, and it can be concluded that the psychological intervention time of the three types of algorithms will have surface contribution differences in outliers. In the time of psychological rehabilitation, there will be a long time, which is a long-term process, and it is impossible to recover in a short time. Compared with the other two algorithms, the deep forest algorithm has shorter overall running time, higher detection efficiency, perfect accuracy, and better overall node mining quality.

### Constructing the Psychological Intervention Mechanism Based on Forest Algorithm

**FIGURE 6:**
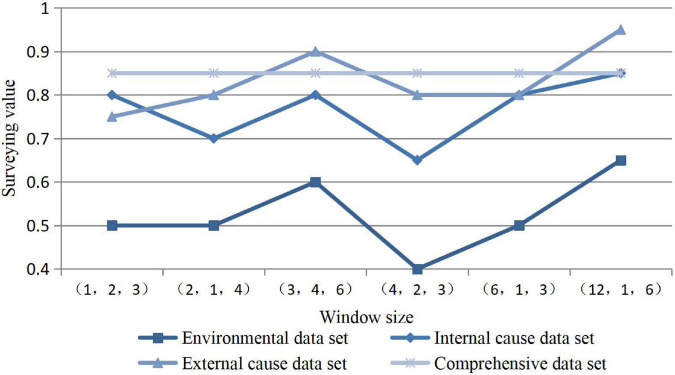
Prediction results of the window size data set.

The performance of psychological parameters can have an impact on the evaluation. Based on the previous window size experiment, we investigated the outlier mining technology. Among them, the window size is defined under the condition of 12 dimensions, such as (3, 4, 6), (12, 1, 6), (6, 1, 3), and other window sizes. It selects a special data set window size to expand the experiment, and the results are as follows in Figure 6.

In the case of large and small data sets under special windows, the influence of environmental factors is not great, because the modern economic environment has entered a good development situation and will not change too much. Especially in the window size (4, 2, 3), the test value is the lowest, indicating that the data set under this window is not very important for this credibility.

#### Experiment on Decision-Making Skills of Psychological Intervention

**FIGURE 7:**
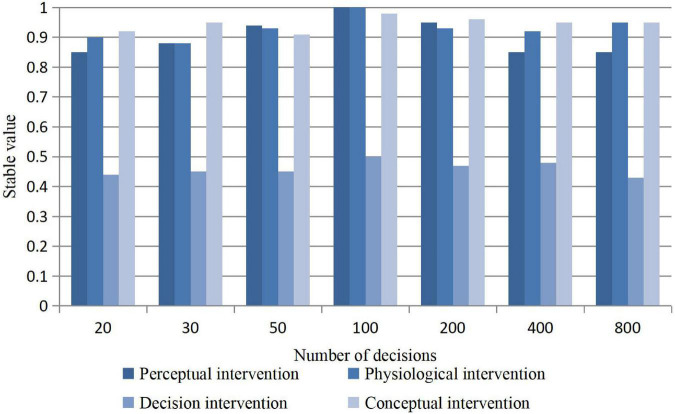
Influence of the number of decisions on the stability of intervention.

Under the same window parameters, the decision number of different intervention methods is analyzed and experimented. The purpose is to explore whether the intervention mechanism of forest algorithm in different data sets is effective. With the increase of the number of decisions, whether the prevention effect is effective is an important assessment point. Because the algorithm consumes too much time and space and increases the load of the system, it should be explored that the best number of decisions is the experimental result of this algorithm. The impact of the number of decisions on intervention is shown in Figure 7.

It can be seen from the figure that, when the number is 100, the system has the highest stability for psychological intervention, which can effectively prevent the early behavior in time. This is also because the algorithm may bring great pressure when the number is too large, and the preventive measures may not be in place in time, which leads to the reduction of prediction probability. When the number is 100, it is the most efficient calculation value of this algorithm, and when the number is 200, 400, and 800, it will only reduce the prediction accuracy.

### Construction of the Psychological Intervention Mechanism in Prevention of Anomie Behavior

**FIGURE 8:**
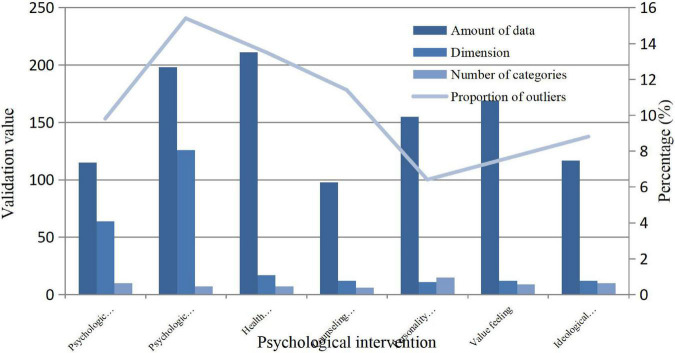
The intervention mechanism data set.

Based on the psychological impact of anomie before the law is affected by many factors, the construction of the intervention mechanism is particularly important. The preliminary test data were tested in order to better carry out rehabilitation, maintenance and other work. Its mechanism operation is based on the system construction functions of data preprocessing, outlier detection, and result analysis. The whole performance verification results and analysis are as follows in Figure 8.

From the intervention conditions of the whole algorithm construction, it can be seen that the early ideological education is an important stage to stifle illegal education so that people can understand the severe punishment of the law and be law-abiding citizens from the heart. Under a large number of data tests and different intervention conditions, it can be clearly seen that personality characteristics can be cultivated in the later stage so that people with noble moral quality can have the ability to distinguish right from wrong.

## Conclusion

Nowadays, under the situation that everyone is equal before the law, people strictly abide by the social order. However, there are some undesirable elements who commit crimes, and their hearts do not establish a good sense of behavioral norms, which leads to improper behaviors that are not conducive to social order. Therefore, before the occurrence of this behavior, we should control and eliminate the psychology in time, predict related activities and behaviors, and prevent the occurrence of improper behavior. In this paper, data mining technology in deep learning is used to predict anomie behavior in advance, and relevant measures are taken to improve education. It introduces correlation factors to improve the accuracy of prediction so as to accurately identify the changes of psychological activities. The results of this study are as follows: (1) According to the development of psychological intervention activity mechanism, it is judged that the accurate value of the algorithm is obviously different under different dimensions, and the measured value is the lowest under the condition of Dimension 9. (2) The forest algorithm uses the data transformation process in the window mode to analyze the relevant influence data sets, and obtains the probability distribution result of window size. (3) Compared with the other two algorithms, the forest algorithm has high efficiency in the process of data mining, and its running time is relatively low. (4) In the whole prediction intervention process, it will be affected by the eigenvalue of each node process, and the results will be obviously different under different intervention conditions. Further research work in the future will mainly study the evolution of legal issues under the background of new technologies and explore the legitimacy of the application of deep learning or machine learning methods under the legal framework.

Shortcomings and prospects: (1) In the face of a large number of data combination, the complex algorithm of deep learning in time and space is extremely demanding, which leads to the reduction of the efficiency of each outlier detection. (2) When seeking a suitable parameter to express, the stability of the algorithm will appear ideal effect, which is beneficial to the next work. (3) When the input characteristic parameters are relatively special, it is necessary to carry out relevant optimization treatment and then carry out the next stage test. (4) Compared with the data set construction of related educational knowledge, in fact, people’s own values are relatively important, which is also one of the subjective elements

## Data Availability Statement

The original contributions presented in the study are included in the article/supplementary material, further inquiries can be directed to the corresponding author.

## Author Contributions

The author confirms being the sole contributor of this work and has approved it for publication.

## Conflict of Interest

The author declares that the research was conducted in the absence of any commercial or financial relationships that could be construed as a potential conflict of interest.

## Publisher’s Note

All claims expressed in this article are solely those of the authors and do not necessarily represent those of their affiliated organizations, or those of the publisher, the editors and the reviewers. Any product that may be evaluated in this article, or claim that may be made by its manufacturer, is not guaranteed or endorsed by the publisher.

## References

[B1] Chen G. Y. Zhang J. B. Meng Y. Tao M. C. Li Z. M. Qian L. (2013). Investigation on the psychological crisis intervention needs of public emergencies in China. *China Health Educ.* 30 492–495.

[B2] He Y. F. Zhang L. Xi X. L. (2020). Focus psychological intervention on exercise ability, lung function and self-management ability of elderly patients with chronic obstructive pulmonary disease. *Clin. J. Pract. Hosp.* 17 75–78. 10.3969/j.issn.1672-6170.2020.04.023

[B3] Huang L. P. Zhao N. Zheng Q. L. (2013). Study on mental health status and intervention effect of occupational disease patients. *China Occup. Med.* 40 195–199.

[B4] Huang Z. H. Xi C. (2018). Investigation on mental health status of community medical staff and evaluation of intervention effect under materialized situation. *Guangzhou Med.* 49 63–66.

[B5] Jiang Q. F. Wang W. P. Hu M. Y. (2013). Effect of comprehensive psychological intervention on psychological behavior of patients with depression. *China Public Health* 29 878–880. 10.15912/j.cnki.gocm.2018.09.203

[B6] Jin Y. (2016). Analysis of the connection between violation of public security administration and criminal offence CPS. *J. Chin. People’s Public Security Univ.* 5 64–72.

[B7] Li C. Shang Y. P. (2017). Research on the influence of academic environment on graduate students’ academic anomie behavior-taking business administration major as an example CPS. *J. Pingdingshan Univ.* 32 119–124.

[B8] Li X. C. (2019). Research on Youth’s Network Moral Anomie and Countermeasures. *Rural Econ. Technol.* 30 239–240. 10.3969/j.issn.1008-6609.2013.01.031

[B9] Liang Y. Lin Y. (2016). Effect of health education on self-perceived burden and quality of life of elderly patients with chronic obstructive pulmonary disease. *China Health Educ.* 32 1036–1039. 10.16168/j.cnki.issn.1002-9982.2016.11.021

[B10] Pan L. M. (2015). Sociological analysis of college students’ network moral anomie. *J. Hubei Corr. Univ.* 28 46–47. 10.3969/j.issn.1671-5918.2015.02-023

[B11] Pan X. S. (2017). On the regulation and supervision of advertising in We Media. *J. Beijing Univ. Posts Telecom.* 19 33–38. 10.3969/j.issn.1008-7729.2017.02.005

[B12] Shi X. Lu P. Y. (2018). Overview of the research on college students’ network moral anomie CPS. *J. Hubei Open Vocat. Coll.* 31 69–70. 10.3969/j.issn.1671-5918.2018.22.032

[B13] Sun G. X. (2017). Research on the subordination and independence of administrative crime’s illegality judgment. *Jurist* 1 48–62.

[B14] Sun J. T. Tang Y. Li X. D. (2014). Effect of psychological intervention on dyspnea in elderly COPD patients. *Chin. J. Gerontol.* 34 6206–6207. 10.3969/j.issn.1005-9202.2014.21.129

[B15] Sun X. M. Ren X. D. Cao Y. X. (2016). Effect of group psychological counseling combined with peer education on anxiety and depression of pregnant women undergoing selective fetal reduction of multiple pregnancies. *Chin. J. Pract. Nurs.* 32 1215–1219. 10.3760/cma.j.issn.1672-7088.2016.16.004 30704229

[B16] Tian Y. Zhou C. (2017). On the influence of scientific research moral anomie in the Haruko incident. *Think Tank Theory Pract.* 2 45–49. 10.19318/j.cnki.issn.2096-1634.2017.06.08

[B17] Wang Y. (2017). An analysis of contemporary college students’ moral anomie. *Sci. Educ. Wenhui* 12 17–18.

[B18] Wen X. L. (2012). Disproof: The Soul Midwifery of Legal Argumentation Procedure Ethics the Significance of Socrates’ Disproof Law. *Chin. Foreign Law* 24 301–311.

[B19] Wu X. C. Zhou X. Lin C. D. Chen J. L. (2015). Study on the influence mechanism and intervention of adolescent post-traumatic psychological reaction CPS. *Psychol. Dev. Educ.* 31 117–127. 10.16187/j.cnki.issn1001-4918.2015.01.16

[B20] Yang J. D. (2017). Research on moral anomie behavior of college students under the perspective of network ideological and political education. *J. Hubei Corr. Univ.* 30 71–72. 10.3969/j.issn.1671-5918.2017.04.036

[B21] Zhang J. C. Sun S. (2013). Psychological injury and prevention of earthquake rescuers CPS. *Dis. Sci.* 28 150–152+159. 10.3969/j.issn.1000-811X.2013.01.031

[B22] Zhao W. J. (2019). The influence of moral anomie of public figures on teenagers’ socialization. *Middle Sch. Polit. Teach. Ref.* 2019 66–68.

[B23] Zhao Y. Gong W. Q. (2016). Investigation on work stress of psychiatrists and observation on intervention effect of group psychological counseling. *J. Psychiatry* 29 273–276. 10.3969/j.issn.2095-9346.2016.04.010

[B24] Zhou M. J. (2019). Research on Moral Anomie of College Students and Its Countermeasures CPS. *Drama House* 24 165–166.

